# CAREGIVER BURDEN AMONG LEBANESE OLDER ADULTS: FINDINGS FROM LSAHA (LEBANON STUDY ON AGEING AND HEALTH)

**DOI:** 10.1093/geroni/igae098.0154

**Published:** 2024-12-31

**Authors:** Sawsan Abdulrahim, Monique Chaaya, Aya El Sammak, Alexandra Abi Nassif, Mayssan Kabalan, Carlos Mendes de Leon

**Affiliations:** American University of Beirut, Cambridge, Massachusetts, United States; American University of Beirut, Beirut, Beyrouth, Lebanon; American University of Beirut, Beirut, Beyrouth, Lebanon; American University of Beirut, Beirut, Beyrouth, Lebanon; American University of Beirut, Beirut, Beyrouth, Lebanon; Georgetown University, Washington, District of Columbia, United States

## Abstract

This study examines correlates of caregiver burden in a sample of older adults (≥60years) in a rural district in Lebanon who provide care to other older adults. Utilizing baseline data from the new Lebanon Study on Ageing and Health (LSAHA), we assessed associations of the Zarit Caregiver Burden Scale with sociodemographic characteristics of the care provider, relationship to care recipient, and type of care provided. Of a total of 1,345 participants, 216 (16%) reported providing care. A higher percentage of women (17%) than men (14%) were caregivers, while a higher percentage of men (47%) than women (37%) provided care to their spouses. After dichotomizing the Caregiving Burden Scale, high burden was more common among women (37%) then men (20%), and among the non-married/widowed (41%) than the married (28%). High burden was lowest among participants with more than 12th grade education. In multivariable logistic regression analysis, high education was significantly associated with lower odds of high burden (OR=0.18; %95 CI=0.04; 0.73) while caregiver gender and marital status were not. Those who provided care for healthcare-related issues were also significantly more likely to report high burden (OR=2.78; 95% CI=1.01; 7.65). In the setting of a mostly rural Lebanese population, the findings point to the burden associated with providing care to other older adults dealing with healthcare issues, possibly due to lack of access or proximity to healthcare facilities. Further analysis will focus on burden associated with characteristics of the care recipient and care provided to others inside versus outside the household.

